# Analysis of the effects of nonextensivity for a generalized dissipative system in the SU(1,1) coherent states

**DOI:** 10.1038/s41598-022-05292-x

**Published:** 2022-01-31

**Authors:** Jeong Ryeol Choi

**Affiliations:** Department of Nanoengineering, Kyonggi University, Yeongtong-gu, Suwon, Gyeonggi-do 16227 Republic of Korea

**Keywords:** Mathematics and computing, Optics and photonics, Physics

## Abstract

The characteristics of nonextensivity for a general quantum dissipative oscillatory system in the SU(1,1) coherent states are investigated using the invariant operator method. We consider a deformed Caldirola-Kanai oscillator represented in terms of a parameter *q* which is a measure of the degree of nonextensivity. The nonextensivity effects on the parametric evolution of the SU(1,1) coherent states are elucidated. We compare our results with those of previous researches and address the advantage of our methodology which adopts the linear invariant operator. In particular, the nonextensive behaviors associated with the fluctuations of canonical variables and the dissipation of quantum energy are analyzed in detail regarding their dependence on *q*. The properties of SU(1,1) coherent states that we adopt here can be utilized in quantum-information processes such as cloning, swapping, and teleportation of state information.

## Introduction

As is well known, Boltzmann-Gibbs (BG) statistics achieved remarkable success, because it provides a standard way of thermostatistical analyses incorporated with ergodic theory. Dynamics of lots of physical systems follows BG statistics. However, it has turned out that the statistical behavior of some dynamical systems and associated phenomena does not follow BG statistics. They include long-range spatial and/or temporal interactions, long-range microscopic memory, and dissipative multifractals^1–3^. This abnormal characteristic originates from nonextensive features of such systems, which require another statistical formalism that covers nonextensivity. Tsallis introduced a generalized thermostatistics with a concept of nonextensive entropy in Ref. 4, which is suitable for describing the mechanism of nonextensivity. Soon after this seminal report, it turned out that Tsallis statistics is very useful in the analyses of overall nonextensive dynamical phenomena, including black-body radiation^5^, gravitation^6^, Euler turbulence^7^, biological evolution^8^, intrinsic inhomogeneities in manganites^9^, nonlinear dynamical dissipation^10^, etc.^11,12^.

Özeren has analyzed the nonextensive properties of a damped oscillator^1^ which is described in terms of a deformed exponential function considering the parametric time evolution of the SU(1,1) coherent states. Glauber coherent states for the deformed damped oscillator driven by an external force has also been studied^13^. For several physical systems, the deviation of the decay of energy or signal from the usual exponential falloff was investigated so far^14–18^. If we combine the mechanism underlying such nonexponential decays with the knowledge of generating typical SU(1,1) coherent states^19,20^, experimental realization of the SU(1,1) coherent states with a nonextensive damping can be achieved.

To study nonextensivity for the dissipative system, Özeren used the destruction and creation operators associated with the simple harmonic oscillator (DCOSHO). Historically, DCOSHO was used in order to derive quantum solutions of harmonic oscillators with time-varying parameters as well as the simple harmonic oscillator. For instance, Tibaduiza *et al.* derived quantum algebraic solutions for nonstationary harmonic oscillators using Lie algebraic approach based on DCOSHO^21,22^.

However, it is also well known that quantum features of such a time-dependent Hamiltonian system are equally well described via other theories that do not use DCOSHO. As one of such alternative theories, the invariant operator theory^23,24^ devised by Lewis and Riesenfeld is a very useful tool in describing quantum characteristics of time-dependent Hamiltonian systems which were firstly treated by Husimi^25^. In fact, the research for quantized time-dependent Hamiltonian systems has achieved great success with the use of the invariant operators. (e.g., see Refs. 26–33 and references there in).

Although the research for the deformed damped oscillator with SU(1,1) coherent states was continued further after the report of Özeren^1^, such research so far was rather focused on treating the system using the formalism of DCOSHO. Inspired by the above-mentioned usefulness of the Lewis-Riesenfeld invariant formalism in the context of time-dependent systems, we investigate in this work the nonextensive dynamics of the SU(1,1) coherent states for the generalized damped harmonic oscillator using a linear invariant operator. To be sure, destruction and creation operators constructed from the invariant operator theory of quantization are in general different from DCOSHO. A set of destruction and creation operators associated with the invariant operator theory (DCOIOT), instead of DCOSHO, will be used. The advantage of the use of DCOIOT in the research of time-dependent harmonic oscillators, such as normal/generalized Caldirola-Kanai (CK) oscillators^34,35^, is that it enables us to obtain exact quantum solutions (without any approximation) so far as the classical solution of the given system is known^31^.

This paper is organized as follows. At first, the invariant operator theory of the generalized CK oscillator is described and the related SU(1,1) generators are constructed. Then the nonextensivity of the SU(1,1) coherent states that are introduced by Perelomov is investigated. and the corresponding results are compared with those of the previous theory which is based on Özeren’s work^1^ and its improved one^36^. Our theory is applied to the analysis of the behavior of nonextensive dissipative oscillatory systems. Lastly, we give the concluding remarks.

## Dynamics of the generalized nonextensive CK oscillator

### Basics of the general CK oscillator with nonextensivity

**Figure 1:**
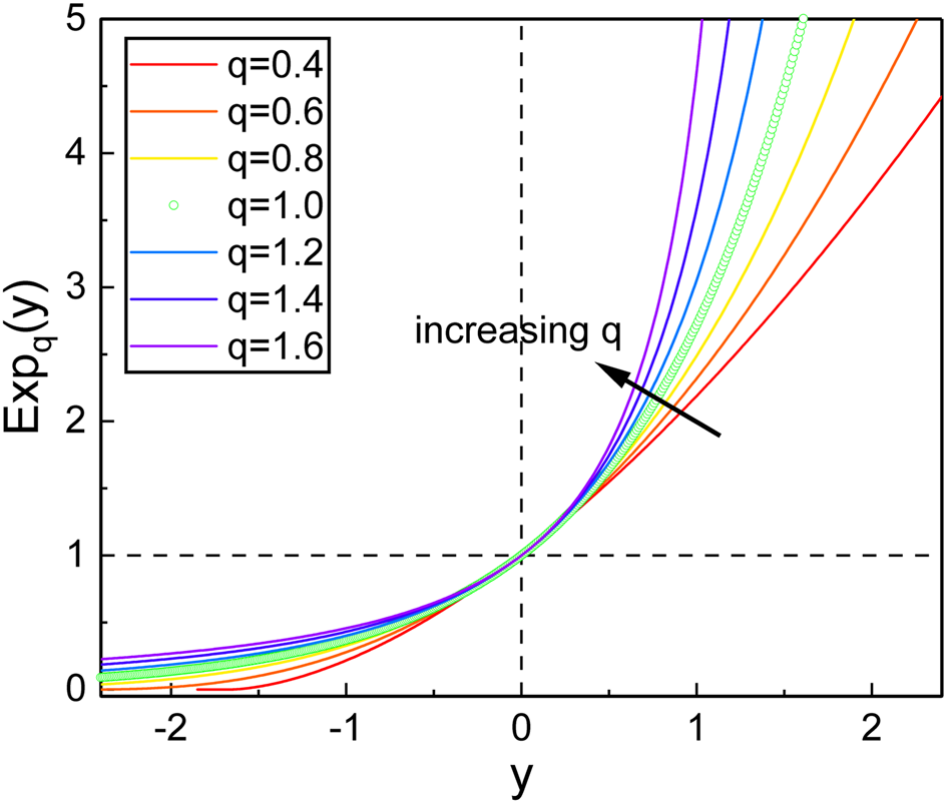
q-exponential function for several different values of *q*.

Various physical systems subjected to a friction-like force which is a linear function of velocity can be modeled by the formal CK oscillator. The Hamiltonian of the CK oscillator is given by^34,35^

1 $$\begin{aligned} {\hat{H}} = e^{-\gamma t} \frac{{\hat{p}}^2}{2m} + \frac{1}{2} e^{\gamma t} m \omega ^2 {\hat{x}}^2, \end{aligned}$$

where $\gamma$ is a damping constant. This Hamiltonian can be generalized by replacing the ordinary exponential function with a deformed one that is defined by^1,37^

2 $$\begin{aligned} \exp _q {(y)} = [1+(1-q)y]^{1/(1-q)}, \end{aligned}$$

with an auxiliary condition

3 $$\begin{aligned} 1+(1-q)y \ge 0, \end{aligned}$$

where *q* is a parameter indicating the degree of nonextensivity. This generalized function is known as the q-exponential and has its own merit in describing non-idealized dynamical systems. The characteristic behavior of the q-exponential function is shown in Fig. 1. In the field of thermostatistics, a generalization of the Gaussian distribution through the q-exponential is known as the Tsallis distribution that is well fitted to many physical systems of which behavior does not follow the usual BG statistical mechanics^38^.

In terms of Eq. (2), we can express the generalized CK Hamiltonian in the form^1^

4 $$\begin{aligned} {\hat{H}}_q = \frac{{\hat{p}}^2}{2m \exp _q{(\gamma t)}} + \frac{1}{2} \exp _q{(\gamma t)} m \omega ^2 {\hat{x}}^2. \end{aligned}$$

This Hamiltonian is Hermitian and, in the case of $q \rightarrow 1$, it recovers to the ordinary CK one that is given in Eq. (1). From the use of the Hamilton’s equations in one dimension, we can derive the classical equation of motion that corresponds to Eq. (4) as

5 $$\begin{aligned} \ddot{x} + \frac{\gamma }{1+(1-q)\gamma t}{\dot{x}} + \omega ^2 x = 0. \end{aligned}$$

In an extreme case where $q \rightarrow 0$, Eq. (2) reduces to a linear function $1+y$. Along with this, Eq. (5) reduces to

6 $$\begin{aligned} \ddot{x} + \frac{\gamma }{1+\gamma t}{\dot{x}} + \omega ^2 x = 0. \end{aligned}$$

If we think from the pure mathematical point of view, it is also possible to consider even the case that *q* is smaller than zero based on the condition given in Eq. (3). However, in most actual nonextensive systems along this line, the value of *q* may not deviate too much from unity which is its standard value. So we will restrain to treating such extreme cases throughout this research.

In general, for time-dependent Hamiltonian systems, the energy operator is not always the same as the given Hamiltonian. The role of the Hamiltonian in this case is restricted: It plays only the role of a generator for the related classical equation of motion. From fundamental Hamiltonian dynamics, we can see that the energy operator of the generalized damped harmonic oscillator is given by^26,39^

7 $$\begin{aligned} {\hat{E}}_{q} = {\hat{H}}_q/\exp _q{(\gamma t)}. \end{aligned}$$

**Figure 2:**
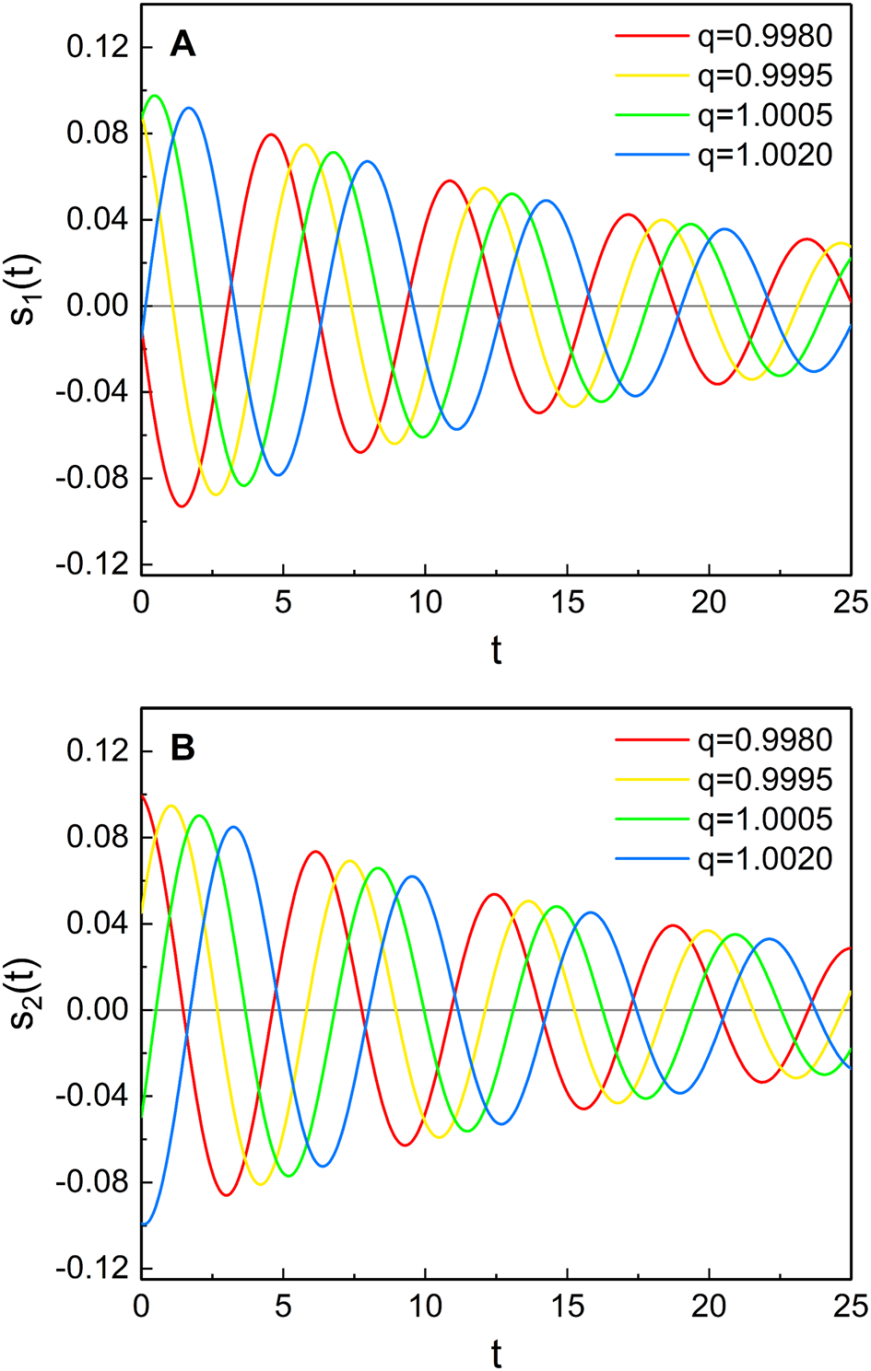
Time evolution of $s_1(t)$ (**A**) and $s_2(t)$ (**B**) for several different values of *q*. We used $\omega =1$, $\gamma =0.1$, and $s_{0,1}=s_{0,2}=0.1$.

Let us denote two linearly independent homogeneous real solutions of Eq. (5) as $s_1(t)$ and $s_2(t)$. Then, from a minor mathematical evaluation, we have^40,41^

8 $$\begin{aligned} s_1(t)= & {} {s}_{0,1}\sqrt{\frac{\pi \omega }{2\gamma (1-q)}} [\exp _q{(\gamma t)}]^{-q/2} J_\nu \left( \frac{\omega }{(1-q)\gamma } + \omega t \right) , \end{aligned}$$

9 $$\begin{aligned} s_2(t)= & {} {s}_{0,2}\sqrt{\frac{\pi \omega }{2\gamma (1-q)}} [\exp _q{(\gamma t)}]^{-q/2} N_\nu \left( \frac{\omega }{(1-q)\gamma } + \omega t \right) , \end{aligned}$$

where $J_\nu$ and $N_\nu$ are the Bessel functions of the first and second kind, ${s}_{0,1}$ and ${s}_{0,2}$ are constants which have dimension of position, and $\nu = {q}/{[2(1-q)]}$. From Fig. 2, we see that the phases in the time evolutions of $s_1(t)$ and $s_2(t)$ are different depending on the value of *q*. Now we can represent the general solution of Eq. (5) in the form

10 $$\begin{aligned} x(t) = c_1 s_1(t) + c_2 s_2(t), \end{aligned}$$

where $c_1$ and $c_2$ are arbitrary real constants.

We introduce another time function *s*(*t*) that will be used later as

11 $$\begin{aligned} s(t) = \sqrt{s_1^2(t)+s_2^2(t)}. \end{aligned}$$

This satisfies the differential equation^42^

12 $$\begin{aligned} \ddot{s}(t) + \frac{\gamma }{1+(1-q)\gamma t}{\dot{s}}(t) + \omega ^2 s(t) - \frac{\Omega ^2}{[m\exp _q{(\gamma t)}]^2} \frac{1}{s^3(t)} = 0, \end{aligned}$$

where $\Omega$ is a time-constant which is of the form

13 $$\begin{aligned} \Omega = m \exp _q{(\gamma t)} [s_1 {\dot{s}}_2 - {\dot{s}}_1 s_2 ]. \end{aligned}$$

By differentiating Eq. (13) with respect to time directly, we can readily confirm that $\Omega$ does not vary in time.

### Invariant operator theory and the SU(1,1) description

In accordance with the invariant operator theory, the invariant operator must satisfy the Liouville-von Neumann equation which is

14 $$\begin{aligned} \frac{d {\hat{I}}}{d t} = \frac{\partial {\hat{I}}}{\partial t} + \frac{1}{i\hbar } [{\hat{I}},{\hat{H}}_q] = 0. \end{aligned}$$

A straightforward evaluation after substituting Eq. (4) into the above equation leads to^24,40^

15 $$\begin{aligned} {\hat{I}} = \hbar \Omega \left( {\hat{b}}^\dagger {\hat{b}} + \frac{1}{2}\right) , \end{aligned}$$

where ${\hat{b}}$ is a destruction operator defined as

16 $$\begin{aligned} {\hat{b}} = \sqrt{\frac{1}{2\hbar \Omega }} \left[ \left( \frac{\Omega }{s(t)} -i m \exp _q{(\gamma t)} {\dot{s}}(t) \right) {\hat{x}} + i s(t) {\hat{p}} \right] , \end{aligned}$$

whereas its hermitian adjoint ${\hat{b}}^\dagger$ is a creation operator. If we take the limit $\gamma \rightarrow 0$, Eq. (16) reduces to that of the simple harmonic oscillator. One can easily check that the boson commutation relation for ladder operators holds in this case: $[{\hat{b}},{\hat{b}}^\dagger ]=1$. This consequence enables us to derive the eigenstates of ${\hat{I}}$ in a conventional way.

The zero-point eigenstate $| 0 \rangle$ is obtained from ${\hat{b}}| 0 \rangle =0$. The excited eigenstates $| n \rangle$ are also evaluated by acting ${\hat{b}}^\dagger$ into $| 0 \rangle$ *n* times. The Fock state wave functions $| \psi _n \rangle$ that satisfy the Schrödinger equation are different from the eigenstates of ${\hat{I}}$ by only minor phase factors which can be obtained from basic relations^24^. However, we are interested in the SU(1,1) coherent states rather than the Fock states in the present work.

The SU(1,1) generators are defined in terms of ladder operators, such that

17 $$\begin{aligned} \hat{{\mathcal {K}}}_0= & {} \frac{1}{2} \left( {\hat{b}}^\dagger {\hat{b}} + \frac{1}{2}\right) , \end{aligned}$$

18 $$\begin{aligned} \hat{{\mathcal {K}}}_+= & {} \frac{1}{2} ({\hat{b}}^\dagger )^2, \end{aligned}$$

19 $$\begin{aligned} \hat{{\mathcal {K}}}_-= & {} \frac{1}{2} {\hat{b}}^2. \end{aligned}$$

From the inverse representation of Eq. (16) together with its hermitian adjoint ${\hat{b}}^\dagger$, we can express ${\hat{x}}$ and ${\hat{p}}$ in terms of ${\hat{b}}$ and ${\hat{b}}^\dagger$. By combining the resultant expressions with Eqs. (17)–(19), we can also represent the canonical variables in terms of SU(1,1) generators as

20 $$\begin{aligned} {\hat{x}}^2= & {} \frac{\hbar s^2}{\Omega } (2\hat{{\mathcal {K}}}_0 + \hat{{\mathcal {K}}}_+ + \hat{{\mathcal {K}}}_-), \end{aligned}$$

21 $$\begin{aligned} {\hat{p}}^2= & {} \frac{\hbar }{s^2} \Bigg [ 2 \left( \Omega + \frac{[m\exp _q(\gamma t)]^2}{ \Omega } s^2{\dot{s}}^2 \right) \hat{{\mathcal {K}}}_0 -\left( \sqrt{\Omega } - \frac{im\exp _q(\gamma t)}{ \sqrt{\Omega }} s{\dot{s}} \right) ^2 \hat{{\mathcal {K}}}_+ \nonumber \\&-\left( \sqrt{\Omega } + \frac{im\exp _q(\gamma t)}{ \sqrt{\Omega }}s{\dot{s}} \right) ^2 \hat{{\mathcal {K}}}_- \Bigg ]. \end{aligned}$$

The substitution of the above equations into Eq. (4) leads to

22 $$\begin{aligned} {\hat{H}}_q = \delta _0(t) \hat{{\mathcal {K}}}_0 + \delta (t) \hat{{\mathcal {K}}}_+ + \delta ^*(t) \hat{{\mathcal {K}}}_- , \end{aligned}$$

where

23 $$\begin{aligned} \delta _0(t)= & {} \frac{\hbar }{s^2} \left( \frac{\Omega }{m\exp _q{(\gamma t)}} + \frac{1}{\Omega } m\exp _q{(\gamma t)} s^2 {\dot{s}}^2 \right) + \frac{\hbar }{\Omega } m\exp _q{(\gamma t)}\omega ^2 s^2 , \end{aligned}$$

24 $$\begin{aligned} \delta (t)= & {} - \frac{\hbar }{2 m\exp _q{(\gamma t)} s^2} \left( \sqrt{\Omega } - i \frac{m\exp _q{(\gamma t)}s{\dot{s}}}{\sqrt{\Omega }} \right) ^2 + \frac{\hbar }{2\Omega } m\exp _q{(\gamma t)} \omega ^2 s^2 . \end{aligned}$$

In accordance with Gerry’s work (see Ref. 43), Eq. (22) belongs to a class of general Hamiltonian that preserves an arbitrary initial coherent state. In the next section, we will analyze the properties of nonextensivity associated with the SU(1,1) coherent states using the Hamiltonian in Eq. (22).

## Analysis of nonextensivity in the SU(1,1) coherent states

### Nonextensive SU(1,1) coherent states based on DCOIOT

The SU(1,1) coherent states for the quantum harmonic oscillator belong to a dynamical group whose description is based on SU(1,1) Lie algebraic formulation. The analytical representation of the SU(1,1) coherent states provides a natural description of quantum and classical correspondence which has an important meaning in theoretical physics. On the experimental side, optical interferometers like radio interferometers that use four-wave mixers as a protocol for improving measurement accuracy are characterized through the SU(1,1) Lie algebra^44,45^.

According to the development of Perelomov^46^, the SU(1,1) coherent states are defined by

25 $$\begin{aligned} | {\tilde{\xi }};k \rangle = \hat{{\mathcal {D}}}(\beta )|{{{\tilde{0}}}} \rangle _k , \end{aligned}$$

where $\hat{{\mathcal {D}}}(\beta )$ is the displacement operator, $|{{{\tilde{0}}}} \rangle _k$ is the vacuum state in the damped harmonic oscillator, and *k* is the Bargmann index of which allowed values are 1/4 and 3/4. The basis for the unitary space is a set of even boson number for $k=1/4$, whereas it is a set of odd boson number for $k=3/4$. Here, the displacement operator is given by

26 $$\begin{aligned} {\hat{D}}(\beta )= & {} \exp \left[ \frac{1}{2} (\beta ^2 \hat{{\mathcal {K}}}_+ - \beta ^{*2} \hat{{\mathcal {K}}}_-) \right] \nonumber \\= & {} e^{{\tilde{\xi }} \hat{{\mathcal {K}}}_+} \exp \{-2\ln [\cosh (|\beta |^2/2)] \hat{{\mathcal {K}}}_0\} e^{-{\tilde{\xi }}^* \hat{{\mathcal {K}}}_-}, \end{aligned}$$

where $\beta$ is the eigenvalue of ${\hat{b}}$ and ${\tilde{\xi }}$ is an SU(1,1) coherent state parameter of the form

27 $$\begin{aligned} {\tilde{\xi }} = \frac{\beta ^2}{|\beta |^2} \tanh (|\beta |^2/2). \end{aligned}$$

The above equation means that $|{\tilde{\xi }}| <1$. For $k=3/4$ among the two allowed values, the resolution of the identity in Hilbert space is given by^47^

28 $$\begin{aligned} \int d\mu ({\tilde{\xi }};k) | {\tilde{\xi }} ; k \rangle \langle {\tilde{\xi }} ; k| = \mathbf{1}, \end{aligned}$$

where $d\mu ({\tilde{\xi }};k)=[(2k-1)/\pi ] d^2 {\tilde{\xi }} /(1-|{\tilde{\xi }}|^2)^2$. More generally speaking, this resolution is valid for $k>1/2$. For a general case where *k* is an arbitrary value, the exact resolution is unknown. Brif *et al.*, on one hand, proposed a resolution of the identity with a weak concept in this context, which can be applicable to both cases of $k>1/2$ and $k<1/2$^47^. In what follows, various characteristics of the damped harmonic oscillator with and without deformation in quantum physics, such as quantum correlation, phase coherence, and squeezing effect, can be explained by means of the SU(1,1) Lie algebra and the coherent states associated with this algebra^48,49^.

The expectation values of SU(1,1) generators in the states $| {\tilde{\xi }};k \rangle$ are^50^

29 $$\begin{aligned} \langle {\tilde{\xi }} ;k | \hat{{\mathcal {K}}}_0|{\tilde{\xi }};k\rangle= & {} k \frac{1+|{\tilde{\xi }}|^2}{1-|{\tilde{\xi }}|^2 } , \end{aligned}$$

30 $$\begin{aligned} \langle {\tilde{\xi }} ;k | \hat{{\mathcal {K}}}_+|{\tilde{\xi }};k\rangle= & {} \frac{2k{\tilde{\xi }}^*}{1-|{\tilde{\xi }}|^2 } , \end{aligned}$$

31 $$\begin{aligned} \langle {\tilde{\xi }} ;k | \hat{{\mathcal {K}}}_-|{\tilde{\xi }};k\rangle= & {} \frac{2k{\tilde{\xi }}}{1-|{\tilde{\xi }}|^2 }. \end{aligned}$$

Using the above equations, the expectation values of the Hamiltonian given in Eq. (22) are easily identified as^50,51^

32 $$\begin{aligned} {{\mathcal {H}}}_{q,k}= & {} \langle {\tilde{\xi }} ;k | {\hat{H}}_q |{\tilde{\xi }};k \rangle \nonumber \\= & {} \frac{k}{1-|{\tilde{\xi }}|^2} \{ \delta _0(t)(1+|{\tilde{\xi }}|^2) +2 [\delta (t){\tilde{\xi }}^*+\delta ^*(t){\tilde{\xi }}] \} . \end{aligned}$$

To derive the classical equation of motion for ${\tilde{\xi }}$, we introduce the Euler-Langrange equation that is given by^50^

33 $$\begin{aligned} \dot{{\tilde{\xi }}} = \frac{1}{i\hbar }[{\tilde{\xi }}, {{\mathcal {H}}}_{q,k}], \end{aligned}$$

where [*A*, *B*] is a generalized Poisson bracket:

34 $$\begin{aligned} {[}A,B]= \frac{(1- |{\tilde{\xi }}|^2)^2}{2k} \left( \frac{\partial A}{\partial {\tilde{\xi }}} \frac{\partial B}{\partial {\tilde{\xi }}^*} - \frac{\partial A}{\partial {\tilde{\xi }}^*} \frac{\partial B}{\partial {\tilde{\xi }}} \right) . \end{aligned}$$

By evaluating Eq. (33) with the use of Eq. (32), we have

35 $$\begin{aligned} \hbar \dot{{\tilde{\xi }}} = -i \delta _0 (t) {\tilde{\xi }} - i\delta ^* (t){\tilde{\xi }}^2 -i \delta (t). \end{aligned}$$

To analyze this equation, let us divide ${\tilde{\xi }}(t)$ into real and imaginary parts such that

36 $$\begin{aligned} {\tilde{\xi }}(t) = {\tilde{\xi }}_1(t) + i{\tilde{\xi }}_2(t), \end{aligned}$$

where ${\tilde{\xi }}_1(t)$ and ${\tilde{\xi }}_2(t)$ are real. Then, the real and imaginary parts of Eq. (35) can be easily identified, respectively, as

37 $$\begin{aligned} \hbar \dot{{\tilde{\xi }}}_1= & {} \delta _0 {\tilde{\xi }}_2 + 2\delta _1 {\tilde{\xi }}_1{\tilde{\xi }}_2 -\delta _2({\tilde{\xi }}_1^2-{\tilde{\xi }}_2^2) +\delta _2 , \end{aligned}$$

38 $$\begin{aligned} \hbar \dot{{\tilde{\xi }}}_2= & {} -\delta _0 {\tilde{\xi }}_1 -\delta _1({\tilde{\xi }}_1^2-{\tilde{\xi }}_2^2 ) -2\delta _2 {\tilde{\xi }}_1{\tilde{\xi }}_2 -\delta _1 , \end{aligned}$$

where $\delta _1$ and $\delta _2$ are the real and imaginary parts of $\delta (t)\,(\delta=\delta_1 + i\delta_2)$, which are given by

39 $$\begin{aligned} \delta _1= & {} -\frac{\hbar \Omega }{2m\exp _q{(\gamma t)} s^2} + \frac{\hbar }{2\Omega } m\exp _q{(\gamma t)} ({\dot{s}}^2 +\omega ^2 s^2), \end{aligned}$$

40 $$\begin{aligned} \delta _2= & {} \hbar {{\dot{s}}}/{s}. \end{aligned}$$

Note that our results, Eqs. (37) and (38), are different from those of the work of Özeren, i.e., Ref. 1. The essential difference between the two theories is that our theory is based on DCOIOT, whereas Özeren’s work is based on DCOSHO. For convenience, we provide Özeren’s method with some correction of errors in “Methods” section (the last section).

In the above description, $\delta$ involves an imaginary part (namely, $\delta _2$) as well as the real one. In order to see the physical meaning of the $\delta _2$ term, let us consider the limit $\gamma \rightarrow 0$ that the system reduces to the simple harmonic oscillator. In this limit, we can re-choose the two classical solutions of Eq. (5) as sinusoidal forms such that $s_1(t) = s_0 \cos (\omega t + \theta )$ and $s_2(t) = s_0\sin (\omega t + \theta )$. Then *s*(*t*) defined in Eq. (11) reduces to $s_0$ which is a constant. In that case, $\delta _2$ vanishes because it is represented in terms of the time derivative of *s*(*t*). From this, we can conclude that $\delta _2$ means the deviation of the system from the simple harmonic oscillator. Alternatively, this also means how the system is nonstationary. The larger the value of $\delta _2$, the greater the variation of the amplitude of the oscillator. However, for the case that the system is described by the framework of DCOSHO, such an imaginary term does not appear at all times (see Refs. 1,36,49).

It may be possible to evaluate numerical solutions of ${\tilde{\xi }}_1$ and ${\tilde{\xi }}_2$ from Eqs. (37) and (38) within a finite interval of time with the help of the Mathematica program (Wolfram Research), as has been done in Özeren’s work^1^. However, the associated calculation is not an easy task in this case due to the complexity of Eqs. (37) and (38) with Eqs. (23), (39), and (40). Hence, to obtain the explicit value of ${\tilde{\xi }}$, it may be better to apply another method which would be easier. In the next subsection, we will describe an alternative method using the development given here as the bridge needed to do the basis transformation.

### Alternative method for the nonextensivity analysis

As mentioned in the above subsection, it may be favorable to use an alternative method for further development. As such a method, we derive ${\tilde{\xi }}$ from Eq. (27) with the explicit formula of the eigenvalue $\beta$. Note that $\beta$ can be obtained by solving the eigenvalue equation

41 $$\begin{aligned} {\hat{b}} | \beta \rangle = \beta | \beta \rangle . \end{aligned}$$

As a matter of fact, the states $| {\tilde{\xi }};k \rangle$ in Eq. (25) are generalization of $| \beta \rangle$, which were established according to a general scheme designated in Ref. 46. Hence, the states $| {\tilde{\xi }};k \rangle$ retain most of the characteristics of the basic state $| \beta \rangle$. The coherent state $| \beta \rangle$ with the eigenvalue $\beta$ is considered as the zero-point energy state displaced from the origin in phase space by an amount $|\beta |$. Although both the states $| \beta \rangle$ and $| {\tilde{\xi }};k \rangle$ are normalized, they are non-orthogonal and overcomplete. Over completeness means that a system is described by redundant states that spans Hilbert space. From expansion of the system in such states, we can attain its classical features as far as quantum mechanics allows.

Through the use of Eq. (16), we can easily evaluate Eq. (41), leading to the exact formula of $\beta$:

42 $$\begin{aligned} \beta = \sqrt{\frac{1}{2\hbar \Omega }} \left[ \left( \frac{\Omega }{s(t)} -i m \exp _q{(\gamma t)} {\dot{s}}(t) \right) {x}(t) + i s(t) {p}(t) \right] , \end{aligned}$$

where *x*(*t*) and *p*(*t*) are classical solutions of coordinate and momentum, respectively. Whereas *x*(*t*) is given in Eq. (10), *p*(*t*) is derived from the simple relation $p(t) = m \exp _q{(\gamma t)} {\dot{x}}(t)$ provided that *x*(*t*) is known. Thus, Eq. (42) becomes

43 $$\begin{aligned} \beta= & {} \frac{1}{\sqrt{2\hbar (s_1^2 + s_2^2)}} \Bigg \{ \sqrt{\Omega } (c_1 s_1 + c_2 s_2) \nonumber \\&+ i \frac{m \exp _q{(\gamma t)}}{\sqrt{\Omega }} [(s_1^2+ s_2^2)(c_1 {\dot{s}}_1 + c_2 {\dot{s}}_2) -(s_1 {\dot{s}}_1+s_2{\dot{s}}_2)(c_1 s_1 + c_2 s_2)] \Bigg \} \nonumber \\\equiv & {} \beta _{\mathrm{R}} + i \beta _{\mathrm{I}}, \end{aligned}$$

where $\beta _{\mathrm{R}}$ and $\beta _{\mathrm{I}}$ are real and imaginary parts, respectively.

At this stage, it may be useful to put $\beta (t)$ in the form

44 $$\begin{aligned} \beta (t) = \beta _0 e^{i \varphi (t)}, \end{aligned}$$

where

45 $$\begin{aligned} \beta _0= & {} \sqrt{\beta _{\mathrm{R}}^2 + \beta _{\mathrm{I}}^2}, \end{aligned}$$

46 $$\begin{aligned} \varphi (t)= & {} \tan ^{-1} \frac{ \beta _{\mathrm{I}}}{ \beta _{\mathrm{R}}}. \end{aligned}$$

A minor evaluation after substituting the expressions of $\beta _{\mathrm{R}}$ and $\beta _{\mathrm{I}}$ into Eq. (45) leads to

47 $$\begin{aligned} \beta _0 = \sqrt{\frac{\Omega }{2\hbar } (c_1^2+c_2^2)}. \end{aligned}$$

This outcome shows that $\beta _0$ is a constant and represented in terms of $c_1$ and $c_2$. Because $c_1$ and $c_2$ are related to the amplitude of the classical solution as can be seen from Eq. (10), $\beta _0$ ($=|\beta |$) determines the amplitude of the oscillation of the system. If we think of the fact that ${\tilde{\xi }}$ defined in Eq. (27) is expressed only in terms of $\beta$, ${\tilde{\xi }}$ is deeply related to the oscillatory factors of the system such as the amplitude, frequency, and phase. Since the direct differentiation of Eq. (46) with respect to time results in

48 $$\begin{aligned} \frac{d \varphi }{dt} = - \frac{\Omega }{s^2 m \exp _q{(\gamma t)}}, \end{aligned}$$

it is possible to write $\varphi$ in the form

49 $$\begin{aligned} \varphi (t) = - \left( \Omega \int _0^t \frac{dt'}{s^2(t')m\exp _q(\gamma t')} + \varphi _0 \right) , \end{aligned}$$

where $\varphi _0$ is a constant phase.

Now, by substituting Eq. (44) into Eq. (27), ${\tilde{\xi }}$ becomes

50 $$\begin{aligned} {\tilde{\xi }} ={\tilde{\xi }}_0 e^{2i\varphi (t)}, \end{aligned}$$

where ${\tilde{\xi }}_0 = \tanh (\beta _0^2/2)$. If we consider Eq. (47), ${\tilde{\xi }}_0$ does not depend on time. Thus, we confirm that the real and the imaginary parts of ${\tilde{\xi }}$ are just given by

51 $$\begin{aligned} {\tilde{\xi }}_1(t)= & {} {\tilde{\xi }}_0 \cos [2\varphi (t)] , \end{aligned}$$

52 $$\begin{aligned} {\tilde{\xi }}_2(t)= & {} {\tilde{\xi }}_0 \sin [2\varphi (t)] . \end{aligned}$$

where the evolution of the corresponding phase follows Eq. (49). Thus we obtained explicit forms of ${\tilde{\xi }}_i(t) (i=1,2)$.

**Figure 3:**
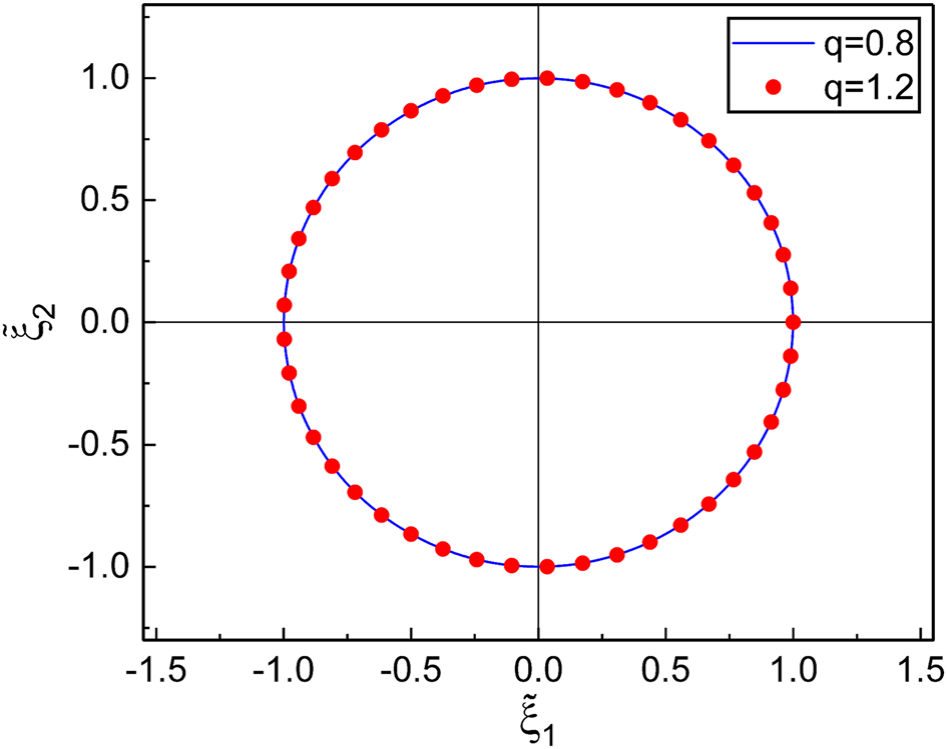
Parametric plot of ${\tilde{\xi }}(t)$ (i.e., ${\tilde{\xi }}_1(t)$ and ${\tilde{\xi }}_2(t)$) on the basis of Eqs. (51) and (52), which are developed using DCOIOT, for two different values of *q* during a cycle of evolution. We used $\omega =1$, $\gamma =0.1$, $m=1$, $\hbar =1$, $c_1=c_2=10$, $s_{0,1}=s_{0,2}=1$, and $\varphi _0=0$; This choice of parameters gives the initial condition as (${\tilde{\xi }}_0$, $\varphi (0)$)$=$(1, 0). This graphic can also be drawn from a parametric numerical evaluation using Eqs. (37) and (38) under the choice of the initial condition as $({\tilde{\xi }}_1(0), {\tilde{\xi }}_2(0))=(1, 0)$.

**Figure 4:**
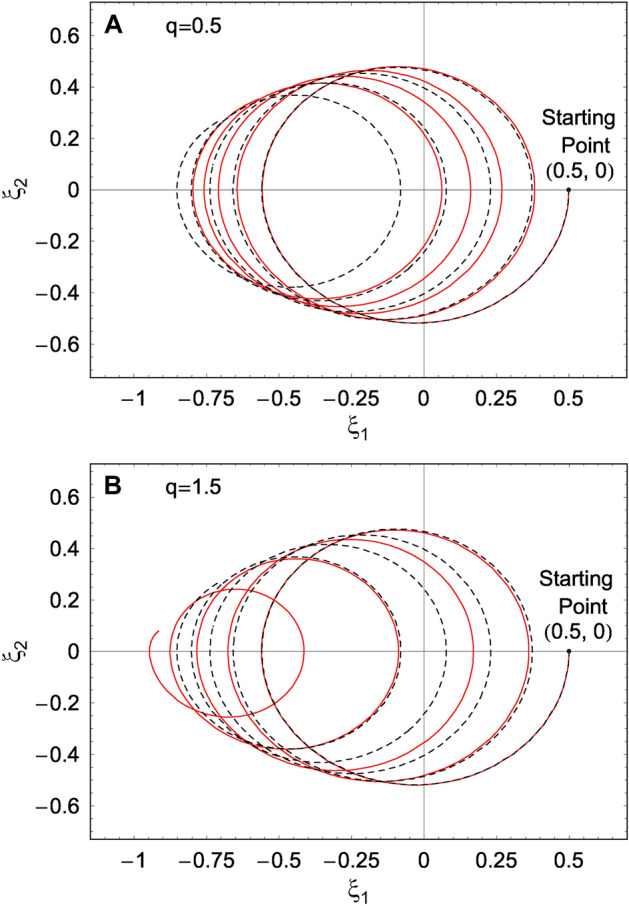
Parametric plot of $\xi (t)$ (i.e., $\xi _1(t)$ and $\xi _2(t)$) which are developed using DCOSHO. This is plotted using the real and imaginary parts of Eq. (61) in “Methods” section, under a low-damping limit within the time interval ($t_{\mathrm{i}}$, $t_{\mathrm{f}}$)$=$ (0, 15). If the magnitude of the damping factor is sufficiently high, the parametric behavior of $\xi (t)$ is quite different from this (see Fig. 2 in Ref. 36). The initial condition we have taken is $\xi _1(0) = 0.5$ and $\xi _2(0) = 0$. The value of *q* for the solid red curve is 0.50 for **A** and 1.50 for **B**. An extra curve (dashed black curve) is added as a reference one which corresponds to the case of $q=1.00$. We used $\omega =1$ and $\gamma =0.1$.

When we analyze the nonextensivity effects of the system, the use of the analytical solutions, Eqs. (51) and (52), may be much more advantageous than the numerical treatment used in Ref. 1. Moreover, the procedure which we employed here in order to derive ${\tilde{\xi }}_i(t)$ is relatively easy and clearer than the evaluation of them using Eqs. (37) and (38). The parametric plot for the evolution of Eqs. (51) and (52) is given in Fig. 3; we confirm that it exhibits a circle of radius ${\tilde{\xi }}_0$. For a comparison purpose, we have also shown the parametric plot of ${\xi }_i(t)$ obtained using the theory of DCOSHO in Fig. 4 on the basis of the evaluation represented in “Methods” section. Remarkably, a compare of Fig. 3 with Fig. 4 shows that the overall appearance of the evolution of the parameter of the SU(1,1) coherent states based on the theory of this work is quite different from the results obtained using the previous theory (with a correction). A characteristic feature of Fig. 4 is that the trajectory is a spiral form whose center proceeds toward the opposite direction of ${{\xi }}_1$, whereas the radius of the spiral decreases in time.

## Applications

The results represented in Eq. (50) (or Eqs. (51) and (52)) can be explicitly implemented in studying diverse nonextensive properties of the system in SU(1,1) coherent states. As examples, we use it in the analyses of variances of the canonical variables and the energy expectation value in the nonextensive SU(1,1) coherent states.

**Figure 5:**
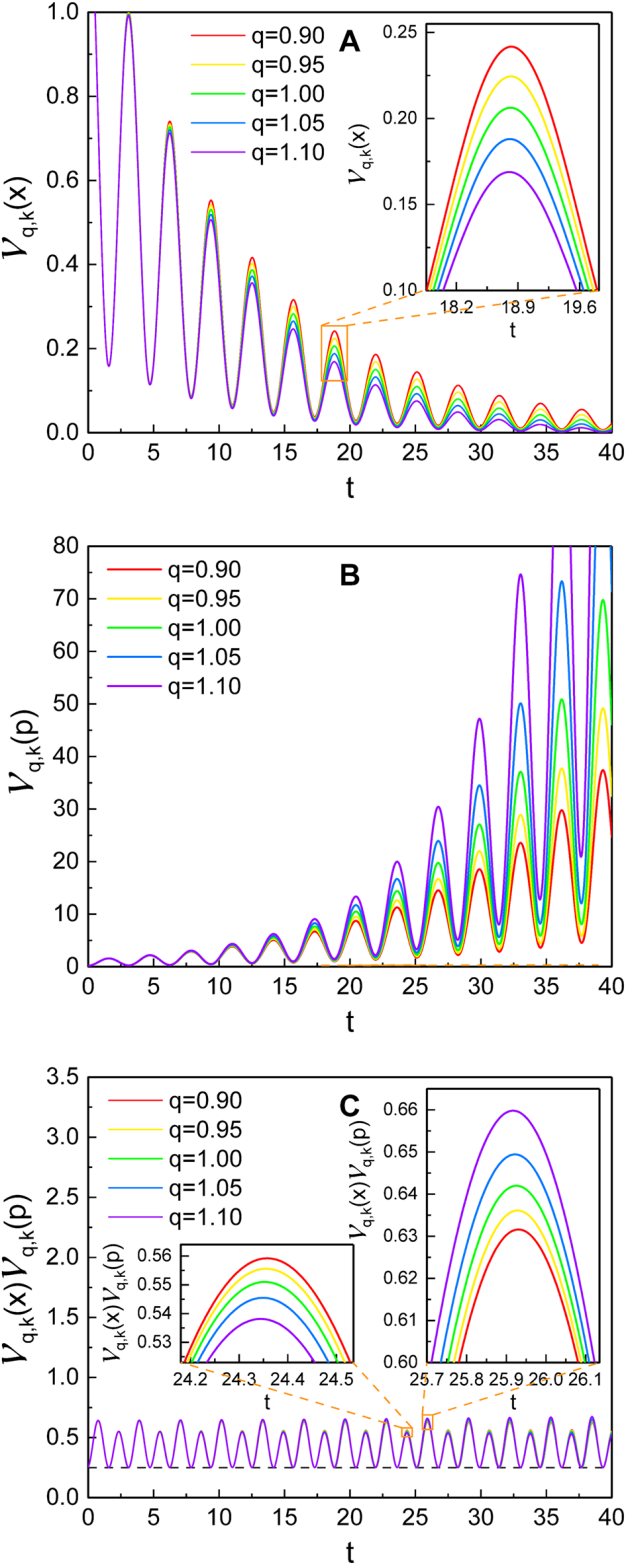
The variances, ${\mathcal {V}}_{q,k}(x)$ (**A**) and ${\mathcal {V}}_{q,k}(p)$ (**B**), and their product (**C**) as a function of *t*, which appear in Eqs. (54) and (55), for several different values of *q*. We used the approximation for $\varphi (t)$, which is given in Eq. (56) (This convention will also be used in the subsequent figures). The insets in **A** and **C** are enlarged graphs for the designated parts. A reference line (dashed black line) on the bottom part of **C** is $\hbar ^2/4$, which is the minimum value allowed as the uncertainty product. We used $\omega =1$, $\gamma =0.1$, $\varphi _0=0$, $m=1$, $\hbar =1$, $c_1=c_2=10$, $k=1/4$, and $s_{0,1}=s_{0,2}=0.1$.

### Variances and uncertainty product

Let us first use our development of the previous sections in investigating the nonextensivity associated with the variances of canonical variables. The variances of any quantum variable ${\hat{A}}$ in the SU(1,1) coherent states are defined as

53 $$\begin{aligned} {{\mathcal {V}}}_{q,k}(A) = \langle {\tilde{\xi }} ;k | {\hat{A}}^2 | {\tilde{\xi }} ;k \rangle -(\langle {\tilde{\xi }} ;k | {\hat{A}} | {\tilde{\xi }} ;k \rangle )^2. \end{aligned}$$

For the case of the canonical variables, the variances can be evaluated by using Eqs. (20) and (21). From a minor evaluation, we easily have

54 $$\begin{aligned} {{\mathcal {V}}}_{q,k}(x)= & {} \frac{2\hbar k s^2}{\Omega (1-{\tilde{\xi }}_0^2)} \{1+{\tilde{\xi }}_0^2 + 2 {\tilde{\xi }}_0 \cos [2\varphi (t)]\}, \end{aligned}$$

55 $$\begin{aligned} {{\mathcal {V}}}_{q,k}(p)= & {} \frac{2\hbar k }{s^2(1-{\tilde{\xi }}_0^2)} \Bigg \{\Omega (1+{\tilde{\xi }}_0^2 - 2 {\tilde{\xi }}_0 \cos [2\varphi (t)]) \nonumber \\&+ \frac{[m \exp _q (\gamma t)]^2}{\Omega }s^2{\dot{s}}^2 (1+{\tilde{\xi }}_0^2 + 2 {\tilde{\xi }}_0 \cos [2\varphi (t)] ) \nonumber \\&+ 4m\exp _q(\gamma t) s {\dot{s}} {\tilde{\xi }}_0 \sin [2\varphi (t)] \Bigg \}. \end{aligned}$$

In the case where the deviation of *q* from unity is not too large, it is possible to approximate Eq. (49) in the form

56 $$\begin{aligned} \varphi (t) \simeq - (\omega _1 t +\varphi _0) , \end{aligned}$$

where $\omega _1$ is a modified frequency which is given by $\omega _1 = \sqrt{\omega ^2 - \gamma ^2/4}$. Under this approximation, we plotted the time evolution of Eqs. (54) and (55), and their product (uncertainty product) in Fig. 5. The red and yellow curves in this figure are the case of super-extensive ($q < 1$), whereas the blue and violet curves are sub-extensive ($q > 1$). The variances for both coordinate and momentum oscillate in time. The envelope of the oscillation of ${{\mathcal {V}}}_{q,k}(x)$ deceases in time while that for ${{\mathcal {V}}}_{q,k}(p)$ increases due to the influence of damping. The variances also exhibit a delicate dependence on *q*: You can see that the variation of ${{\mathcal {V}}}_{q,k}(x)$ is reduced for large *q* at a later time, while the variation of ${{\mathcal {V}}}_{q,k}(p)$ becomes rather drastic in the same situation.

It was shown that quantum states of a damped oscillator correspond to squeezed states^52^. Moreover, any non-adiabatic change in the parameters of a harmonic oscillator produces squeezing. For the case of the nonextensive system given here, squeezing of *x* quadrature grows slightly as *q* increases, whereas squeezing of *p* quadrature reduces at the same situation. This is a manifestation of the emergence of the effect of nonextensivity.

Figure 5C shows a somewhat regular oscillation of the uncertainty product, ${{\mathcal {V}}}_{q,k}(x){{\mathcal {V}}}_{q,k}(p)$, in time. From the first inset in Fig. 5C, we confirm that the amplitude of such an oscillation is slightly smaller when *q* becomes large. On the other hand, the second inset shows an opposite behavior which is that it becomes large as *q* increases. We also see that ${{\mathcal {V}}}_{q,k}(x){{\mathcal {V}}}_{q,k}(p)$ is always larger than or equal to $\hbar ^2/4$, which is the minimum value of the product allowed in quantum mechanics. While the uncertainty product of the two conjugate variables is always non-zero and intrinsic in quantum mechanics, the variation of it (including its components ${{\mathcal {V}}}_{q,k}(x)$ and ${{\mathcal {V}}}_{q,k}(p)$) is novel as a nonclassical effect connected with the nonstationary coherent states.

**Figure 6:**
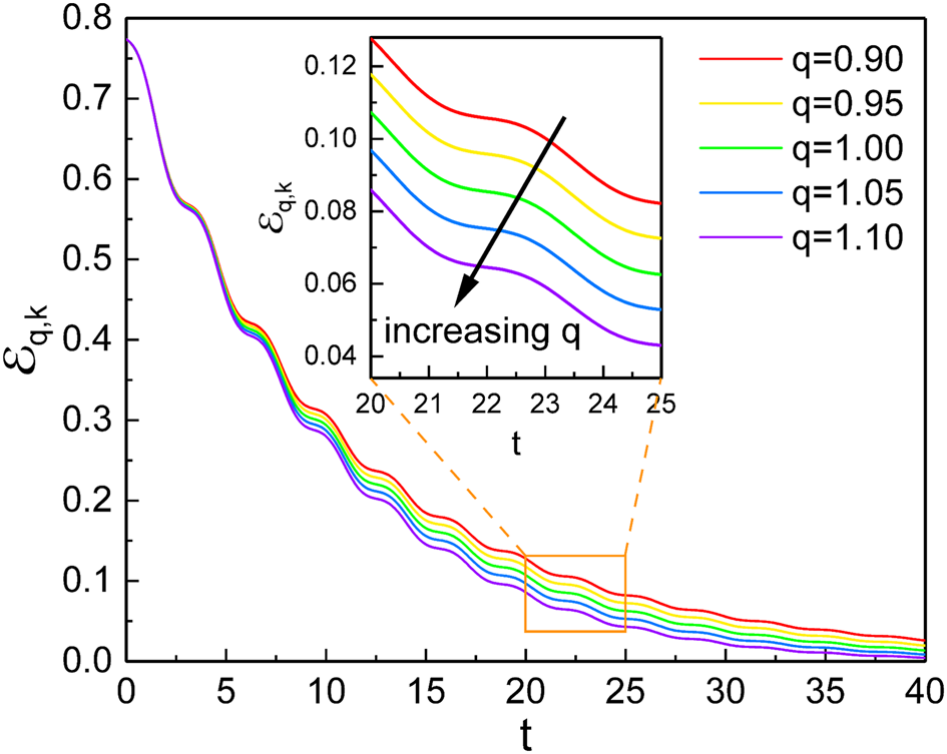
The dependence of the energy expectation value ${{\mathcal {E}}}_{q,k}$ [Eq. (57)] on *q*. We used $\omega =1$, $\gamma =0.1$, $\varphi _0=0$, $m=1$, $\hbar =1$, $c_1=c_2=10$, $k=1/4$, and $s_{0,1}=s_{0,2}=0.1$.

### Quantum energy

Now, we will look at the nonextensivity effects on quantum energy as another application. The expectation values of the energy operator in the SU(1,1) coherent states can be obtained from ${{\mathcal {E}}}_{q,k} = \langle {\tilde{\xi }} ;k | {\hat{E}}_q |{\tilde{\xi }};k \rangle$, where ${\hat{E}}_q$ is given in Eq. (7). A straightforward evaluation of this using Eqs. (22), (32), and (50) results in

57 $$\begin{aligned} {{\mathcal {E}}}_{q,k}= & {} \frac{k}{(1-{\tilde{\xi }}_0^2)\exp _q(\gamma t)} \bigg \{\delta _0(t)(1+{\tilde{\xi }}_0^2)+ 2\hbar {\tilde{\xi }}_0 \bigg [ \bigg ( \frac{m\exp _q(\gamma t)}{\Omega }({\dot{s}}^2 +\omega ^2 s^2) \nonumber \\&-\frac{\Omega }{m\exp _q(\gamma t)s^2} \bigg )\cos [2\varphi (t)] +2\frac{{\dot{s}}}{s} \sin [2\varphi (t)]\bigg ] \bigg \}. \end{aligned}$$

The energy of the oscillator does not dissipate at its turning points in the motion since the velocity at those points is zero. On the other hand, the dissipation of energy is locally maximum when the oscillator acquires the highest velocity at a vicinity of the origin ($x=0$).

**Figure 7:**
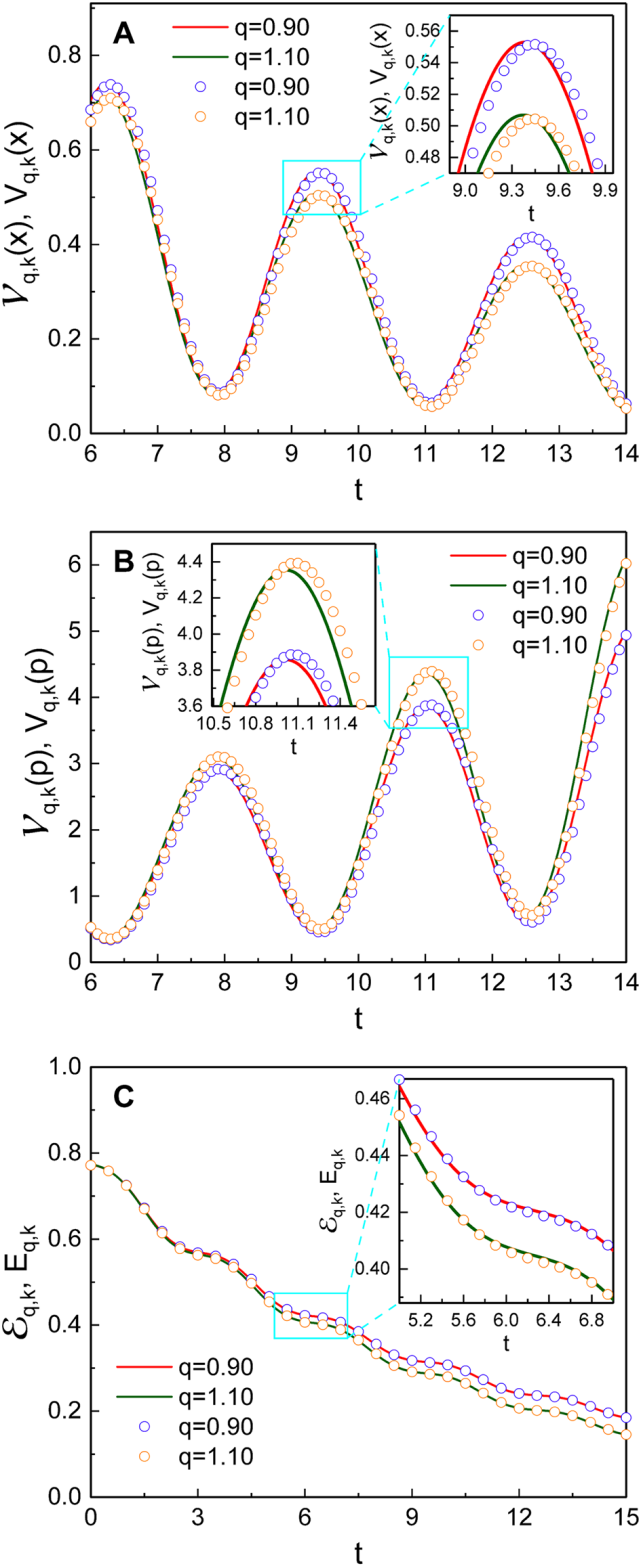
Comparison of the results of this research (solid curves) for ${{\mathcal {V}}}_{q,k}(x)$ (**A**), ${{\mathcal {V}}}_{q,k}(p)$ (**B**), and ${{\mathcal {E}}}_{q,k}$ (**C**) with their counterpart results $V_{q,k}(x)$, $V_{q,k}(p)$, and $E_{q,k}$ in the previous research (circles) performed using DCOSHO. We used $\omega =1$, $\gamma =0.1$, $\varphi _0=0$, $m=1$, $\hbar =1$, $c_1=c_2=10$, $k=1/4$, and $s_{0,1}=s_{0,2}=0.1$. For the case based on DCOSHO, we used Eqs. (62)–(64), where $\xi$ (actually, $\xi _1$ and $\xi _2$) were numerically evaluated from the real and imaginary parts of Eq. (61) with the initial value of $\xi$ as the same value of ${\tilde{\xi }}(0)$: ($\xi _1(0)$, $\xi _2(0)$)=(${\tilde{\xi }}_1(0)$, ${\tilde{\xi }}_2(0)$)= (0.462117, 0.0). Note that ${\tilde{\xi }}_1(0)$ is the same as ${\tilde{\xi }}_0$ in this case, which can be evaluated using Eq. (47).

We see from Fig. 6 that such energy dissipation is more rapid when *q* is large. To understand this consequence, recall from Fig. 1 that the q-exponential function in the region $y>0$ grows faster with *q* when *q* is large. From the analyses performed until now, we can confirm that the effects of nonextensivity are critical to the evolution of the system as a response to a small deviation of the q-exponential from the usual exponential.

**Figure 8:**
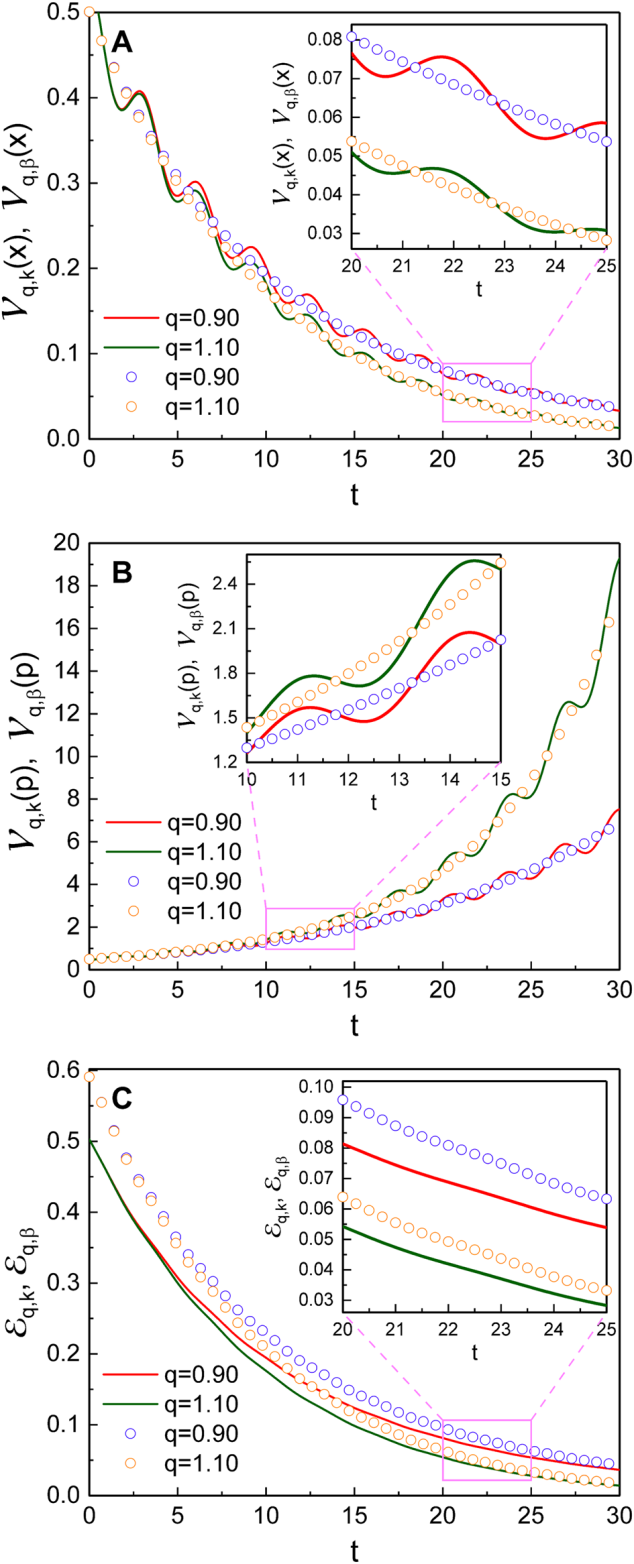
Comparison of our results ${{\mathcal {V}}}_{q,k}(x)$ (**A**), ${{\mathcal {V}}}_{q,k}(p)$ (**B**), and ${{\mathcal {E}}}_{q,k}$ (**C**) with those in the Glauber coherent state, ${{\mathcal {V}}}_{q,\beta }(x)$, ${{\mathcal {V}}}_{q,\beta }(p)$, and ${{\mathcal {E}}}_{q,\beta }$, respectively, for two different values of *q*. Our results are solid curves, whereas the results in the Glauber coherent state are circles. The results associated with the Glauber coherent state, as well as ours, are obtained using DCOIOT. We used $\omega =1$, $\gamma =0.1$, $\varphi _0=0$, $m=1$, $\hbar =1$, $c_1=c_2=0.3$, $k=1/4$, and $s_{0,1}=s_{0,2}=1$.

Figure 7 shows the comparison of our results for variances and an energy expectation value based on using DCOIOT with those of the previous research^36^ based on DCOSHO. You can see the nonextensivity results obtained using DCOSHO in the SU(1,1) coherent states from “Methods” section. Because invariant operator theory admits to obtain complete quantum results which satisfy the Schrödinger equation as mentioned in the introductory part, our outcomes based on DCOIOT are exact. However, Fig. 7 shows that the consequences based on DCOSHO do not deviated so much from our results, especially for the case of the energy expectation value.

**Figure 9:**
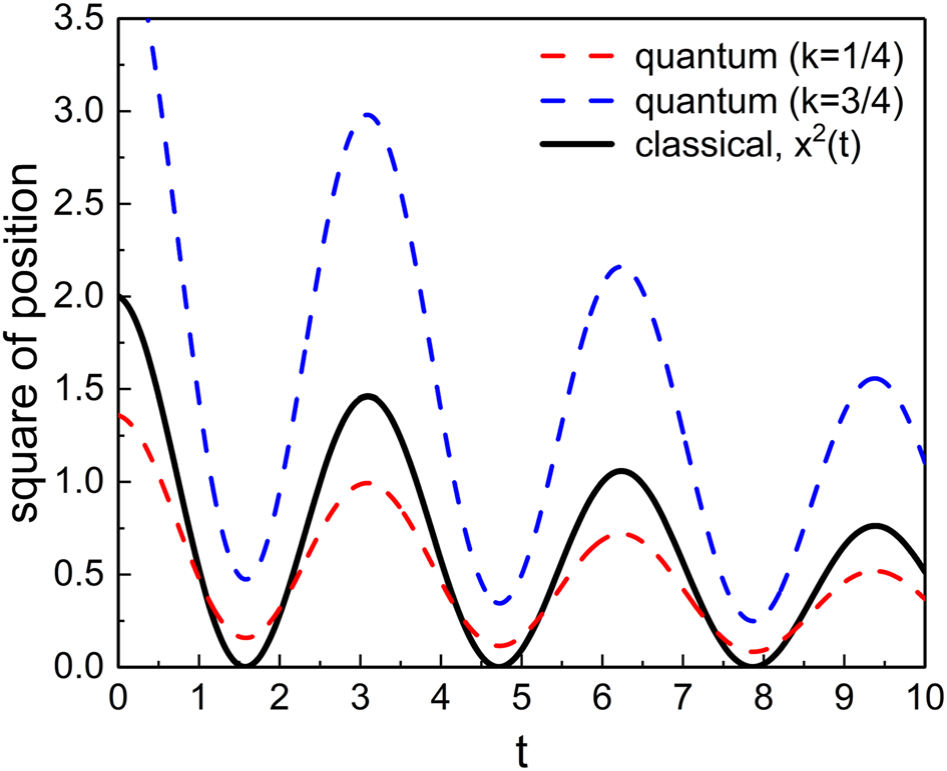
Comparison of the quantum expectation values $\langle {\tilde{\xi }} ;k | {\hat{x}}^2 | {\tilde{\xi }} ;k \rangle$ with the square of the classical position $x^2(t)$, where the allowed values of *k* for quantum results are 1/4 and 3/4 as mentioned previously. We used $q=1.05$, $\omega =1$, $\gamma =0.1$, $\varphi _0=0$, $m=1$, $\hbar =1$, $c_1=c_2=1$, and $s_{0,1}=s_{0,2}=1$.

Figure 8 is the comparison of our results with those^41^ in the Glauber coherent state. The nonextensivity effects in the Glauber coherent state are briefly represented in “Methods” section. While our results for variances ${{\mathcal {V}}}_{q,k}(x)$ and ${{\mathcal {V}}}_{q,k}(p)$ fluctuate over time, ${{\mathcal {V}}}_{q,\beta }(x)$ vary ${{\mathcal {V}}}_{q,\beta }(p)$ in the Glauber coherent state vary monotonically. If we neglect such fluctuations in our results, the time behaviors of the variances, ${{\mathcal {V}}}_{q,k}(x)$ and ${{\mathcal {V}}}_{q,k}(p)$, are nearly the same as those of their counterpart results in the Glauber coherent state. The expectation value of the quantum energy ${{\mathcal {E}}}_{q,k}$ in our research is somewhat smaller than ${{\mathcal {E}}}_{q,\beta }$ in the case where $k=1/4$ (Fig. 8C). However, if we take $k=3/4$ for ${{\mathcal {E}}}_{q,k}$ among its two allowed values, ${{\mathcal {E}}}_{q,k}$ is rather larger than ${{\mathcal {E}}}_{q,\beta }$ (not shown in Fig. 8C).

### Comparison with the classical perspective

We have used the coherent states, Eq. (25), in the previous subsections in order to see variances and quantum energies. It is well known that coherent states resemble classical states as far as quantum mechanics allows. Let us see how the states in Eq. (25) resemble the classical one. To this end, it may be plausible to show that the time evolution of the expectation values $\langle {\tilde{\xi }} ;k | {\hat{x}} | {\tilde{\xi }} ;k \rangle$ in those states are similar to the classical trajectory. However, because the generating functions, Eqs. (17)–(19), are represented in terms of two-photon operators in the terminology of quantum optics, the expectation value of ${\hat{x}}$ in the corresponding states is zero. For this reason, such a scheme is not applicable in the case of SU(1,1) coherent states for an oscillatory system.

Alternatively, we can check that how the time evolution of the expectation value of $${\hat{x}}^2$$ is analogous to the counterpart classical one as a second best. We have provided such a comparison in Fig. 9. This figure shows that the values $$\langle {\tilde{\xi }} ;k | {\hat{x}}^2 | {\tilde{\xi }} ;k \rangle$$ with $$k $$=1/4 and $$k $$=3/4 exhibit oscillatory behaviors with dissipation over time like $$x^2$$ in the classical state. While the local minimum values in the evolution of $$x^2(t)$$ are zero, those of both the two quantum ones are not zero but are some finite values. This quantum consequence is due to the existence of their zero-point quantities^53^ that are intrinsic in quantum mechanics. Such zero-point quantities originate from the Heisenberg’s fundamental uncertainty limit, which is absent in the classical domain.

## Conclusion

The properties of a general nonextensive damped harmonic oscillator have been investigated using the invariant operator theory. Based on the formal developments for the nonextensivity dynamics, we have replaced ordinary exponential function given in the CK Hamiltonian with the q-exponential one. Through this generalization for the formalism of the CK Hamiltonian, it was possible to analyze the nonextensive behavior of the system.

We introduced the destruction and creation operators, ${\hat{b}}$ and ${\hat{b}}^\dagger$, in order to establish the SU(1,1) coherent states. These operators were produced entirely from the invariant operator theory of the deformed system, while the ladder operators used in the work of Özeren^1^ are those which were relevant to the simple harmonic oscillator. The SU(1,1) generators, $\hat{{\mathcal {K}}}_0$, $\hat{{\mathcal {K}}}_1$, and $\hat{{\mathcal {K}}}_2$, were defined in Eqs. (17)–(19) in terms of ${\hat{b}}$ and ${\hat{b}}^\dagger$. To unfold the quantum theory associated with the SU(1,1) coherent states, we have reexpressed the Hamiltonian given in Eq. (4) in terms of these generators [see Eq. (22)].

Based on these procedures, we investigated nonextensive characteristics of the system in connection with the SU(1,1) coherent states. For convenience, we separated the coherent state parameter ${\tilde{\xi }}$ into real (${\tilde{\xi }}_1$) and imaginary (${\tilde{\xi }}_2$) parts. By means of the Euler-Langrange equation, the differential equations associated with the time evolution of ${\tilde{\xi }}_1$ and ${\tilde{\xi }}_2$ were obtained, as shown in Eqs. (37) and (38). However, if we consider the complexity of these equations, it may be somewhat difficult to derive the numerical solutions of ${\tilde{\xi }}_1$ and ${\tilde{\xi }}_2$ from them. Hence, we adopted an alternative method which enabled us to obtain the closed form of the analytical expressions of ${\tilde{\xi }}_1$ and ${\tilde{\xi }}_2$. For this, we have taken the advantage of Eq. (27) with the explicit formula of $\beta (t)$. You can easily confirm the time behavior of ${\tilde{\xi }}_1$ and ${\tilde{\xi }}_2$ from their resulting expressions given in Eqs. (51) and (52).

The effects of nonextensivity with different values of *q* were illustrated in detail and compared with those of Özeren’s work (with some improvement), which have been studied using DCOSHO. The parametric plots of our results for ${\tilde{\xi }}_1$ and ${\tilde{\xi }}_2$ reveal circles (Fig. 3), while those based on the theory developed using DCOSHO reveal spirals (Fig. 4). Figure 5 shows that the variances of *x* and *p* oscillate with time. The envelope of such an oscillation for ${{\mathcal {V}}}_{q,k}(x)$ decreases over time, whereas that for ${{\mathcal {V}}}_{q,k}(p)$ increases. As a manifestation of the nonextensivity, the time variation of ${{\mathcal {V}}}_{q,k}(x)$ becomes small as *q* increases, whereas that of ${{\mathcal {V}}}_{q,k}(p)$ becomes large at the same situation. We confirmed that the energy of this generalized system dissipates over time like in the case of the ordinary damped oscillator, whereas the rate of such a dissipation is slightly higher for larger *q*. If we think of the fact that invariant operator theory gives complete quantum solutions without approximation^31^, our consequences for variances and energy expectation values are exact. By the way, Fig. 7 shows that the counterpart quantum results evaluated on the basis of DCOSHO are not so different from ours. Our results for the variances of the canonical variables and the energy expectation value were also compared with those in the Glauber coherent state, addressing differences and similarities between them. It may be possible to extend our method adopted in this work to any arbitrary form of q-deformed oscillatory systems so long as the system admits SU(1,1) formalism with appropriate SU(1,1) generators.

As a final remark, SU(1,1) coherent states including their general deformed types not only play an important role in traditional quantum optics but are noticeable as an implement of quantum information theory as well, whose main subjects are quantum computation, quantum teleportation, and quantum cryptography^54–56^. The technique of cloning and swapping of (generalized) coherent states based on Lie algebras SU(1,1) and SU(2) are crucial in realizing next generation quantum-information-based technology. In particular, SU(1,1) Lie algebra and its related coherent states can be utilized in general in presenting an algebraic adaptation of Kieu’s hypercomputational quantum algorithm (KHQA)^56,57^ in quantum computation.

## Methods

### Methods summary

We introduce a Hamiltonian of a modified CK oscillator that exhibits nonextensive effects. This Hamiltonian is represented in terms of a nonextensivity parameter *q*. We establish a quadratic invariant operator ${\hat{I}}$ associated with the Hamiltonian from the Liouville-von Neumann equation. The invariant operator is represented in terms of annihilation and creation operators (${\hat{b}}$ and ${\hat{b}}^\dagger$) that obey the commutation relation $[{\hat{b}},{\hat{b}}^\dagger ]=1$. SU(1,1) generators, $\hat{{\mathcal {K}}}_0$, $\hat{{\mathcal {K}}}_+$, and $\hat{{\mathcal {K}}}_-$, are constructed in terms of the annihilation and creation operators according to their definitions. Based on these, the Perelomov’s SU(1,1) coherent states are formulated. Using the wave functions $| {\tilde{\xi }};k \rangle$ in the SU(1,1) coherent states, we evaluate expectation values of canonical variables and their squares. Quantum energy, in addition to the fluctuations of canonical variables and the corresponding uncertainty product, is investigated utilizing such values.

### Özeren’s methods for the approach of nonextensivity

In Ref. 1, Özeren studied nonextensive properties of the generalized damped harmonic oscillator by using a set of DCOSHO. His theory is somewhat improved in Ref. 36. Here, we will briefly describe it with some corrections.

Özeren’s research is based on the well-known destruction operator associated with the simple harmonic oscillator, which is given by ${\hat{a}} = \sqrt{{m\omega }/{(2\hbar )}} {\hat{x}} + ({i}/{\sqrt{2m\omega \hbar }}){\hat{p}},$ and its hermitian adjoint ${\hat{a}}^\dagger$ (the creation operator). The eigenvalue equation for the destruction operator can be written as ${\hat{a}}| \alpha \rangle = \alpha | \alpha \rangle$, where $\alpha$ is the eigenvale and $| \alpha \rangle$ is the eigenstate.

Let us consider the SU(1,1) generators, ${\hat{K}}_0$, ${\hat{K}}_+$, and ${\hat{K}}_-$, defined in the same way with those given in Eqs. (17)–(19), but in terms of ${\hat{a}}$ and ${\hat{a}}^\dagger$ instead of ${\hat{b}}$ and ${\hat{b}}^\dagger$. Then, the Hamiltonian given in Eq. (4) can be written in terms of these generators as^36^

58 $$\begin{aligned} {\hat{H}}_q = 2\hbar \omega \cosh _q (\gamma t) {\hat{K}}_0 +\hbar \omega \sinh _q (\gamma t)({\hat{K}}_+ +{\hat{K}}_-), \end{aligned}$$

where

59 $$\begin{aligned} \cosh _q (y)= & {} \frac{1}{2} \{ \exp _q (y) + [\exp _q (y) ]^{-1} \}, \end{aligned}$$

60 $$\begin{aligned} \sinh _q (y)= & {} \frac{1}{2} \{ \exp _q (y) - [\exp _q (y) ]^{-1} \}. \end{aligned}$$

Note that the Hamiltonian in Eq. (58) is somewhat different from that of the Özeren’s original research given in Eq. (15) of Ref. 1 due to some errors in that work.

The SU(1,1) coherent states in this context are defined as $| {\xi };k \rangle = {\hat{D}}(\alpha )|0 \rangle _k ,$ where $|0 \rangle _k$ is the vacuum state and ${\hat{D}}(\alpha )$ is the displacement operator of the form ${\hat{D}}(\alpha ) = \exp [ (\alpha ^2 {\hat{K}}_+ - \alpha ^{*2} {\hat{K}}_-)/2 ]$. The parameter of the SU(1,1) coherent states is defined as $\xi = ({\alpha ^2}/{|\alpha |^2}) \tanh (|\alpha |^2/2)$. If we adopt the same method used in the case of the DCOIOT-based approach, the classical equation of motion for $\xi$ is given by^36^

61 $$\begin{aligned} {\dot{\xi }} = -i\omega [2\xi \cosh _q (\gamma t) + (1+\xi ^2)\sinh _q (\gamma t)]. \end{aligned}$$

If we put $\xi = \xi _1+i\xi _2$ where $\xi _1$ and $\xi _2$ are real, the real and imaginary parts of the above equation are easily obtained. The parametric evolution of $\xi _1$ and $\xi _2$ for a highly damped case with several different values of *q* was shown in Ref. 36. We have also plotted it in Fig. 4 of this work, but in a low-damping limit, for the comparison purpose with our main result (Fig. 3). For more details along this line, see Ref. 36 together with Ref. 1.

### Approach based on DCOSHO

The approach for nonextensivity in the SU(1,1) coherent states based on the use of DCOSHO are given in Ref. 36. We represent its consequences here for comparison purposes. The variances of the canonical variables are given by

62 $$\begin{aligned} V_{q,k}(x)= & {} \frac{2\hbar k}{m\omega (1-|\xi |^2)} (1+|\xi |^2 + \xi + \xi ^* ), \end{aligned}$$

63 $$\begin{aligned} V_{q,k}(p)= & {} \frac{2m\omega \hbar k}{1-|\xi |^2} (1+|\xi |^2 - \xi - \xi ^* ). \end{aligned}$$

The energy expectation values are represented as

64 $$\begin{aligned} E_{q,k} = \frac{2\hbar \omega k}{(1-|\xi |^2)\exp _{q}{(\gamma t)}} [ (1+|\xi |^2)\cosh _{q} (\gamma t) + (\xi + \xi ^*)\sinh _{q} (\gamma t)] . \end{aligned}$$

### Approach based on DCOIOT in the Glauber coherent state

The Glauber coherent state $|\beta \rangle$ is the eigenstate of ${\hat{b}}$ as is represented in Eq. (41). Quantum dynamics of the system in the Glauber coherent state has been described in Ref. 41. The variance of an operator ${\hat{A}}$ in this state can be defined as ${{\mathcal {V}}}_{q,\beta }(A) = \langle \beta | {\hat{A}}^2 | \beta \rangle -(\langle \beta | {\hat{A}} | \beta \rangle )^2.$ For the case of the canonical variables, the variances result in^41^

65 $$\begin{aligned} {{\mathcal {V}}}_{q,\beta }(x)= & {} s^2 \frac{\hbar }{2 \Omega }, \end{aligned}$$

66 $$\begin{aligned} {{\mathcal {V}}}_{q,\beta }(p)= & {} \frac{\hbar \Omega }{2s^2} [ 1+ (m \exp _q (\gamma t) s {\dot{s}}/\Omega )^2 ]. \end{aligned}$$

On the other hand, the quantum energy expectation value is of the form

67 $$\begin{aligned} {{\mathcal {E}}}_{q,\beta }= \eta _0(t) (2 |\beta |^2 +1) + \eta (t) \beta ^{* 2} +\eta ^*(t) \beta ^2, \end{aligned}$$

where

68 $$\begin{aligned} \eta _0(t)= & {} \frac{\hbar m}{4\Omega } \Bigg ( {\dot{s}}^2 + \omega ^2 s^2 +\frac{\Omega ^2}{m^2[\exp _q(\gamma t)]^2 s^2}\Bigg ), \end{aligned}$$

69 $$\begin{aligned} \eta (t)= & {} \frac{\hbar m}{4\Omega } \Bigg [ \Bigg ({\dot{s}} + i\frac{\Omega }{m \exp _q(\gamma t) s}\Bigg )^2 +\omega ^2 s^2\Bigg ]. \end{aligned}$$

Equations (65), (66), and (67) are used in Fig. 8.
